# HDAC inhibition and acetylation‐driven disulfide‐linked aggregation of TDP43: implications for TDP43 proteinopathies

**DOI:** 10.1002/alz70855_100815

**Published:** 2025-12-23

**Authors:** Sungsu Lim, Yun Kyung Kim, Ae Nim Pae

**Affiliations:** ^1^ Korea Institute of Science and Technology, Seoul, 02792, Korea, Republic of (South); ^2^ Korea Institute of Science and Technology, Seoul, Seoul, Korea, Republic of (South)

## Abstract

**Background:**

TDP43 proteinopathies, including amyotrophic lateral sclerosis (ALS) and frontotemporal lobar degeneration (FTLD), are characterized by the pathological aggregation and mislocalization of TDP43 protein. These aberrant aggregates disrupt cellular functions and contribute to neurodegeneration. However, the molecular mechanisms driving TDP43 aggregation remain unclear. Histone deacetylases (HDACs) regulate protein acetylation, a process implicated in protein aggregation and neurodegenerative diseases. This study aims to elucidate the role of HDAC inhibition and acetylation in TDP43 aggregation, particularly focusing on its mislocalization and oligomerization dynamics.

**Method:**

We established a TDP43‐BiFC (Bimolecular Fluorescence Complementation) cell model to visualize TDP43 oligomerization in living cells. Using this system, we examined the effects of cellular stress activators and HDAC inhibitors on TDP43 aggregation. The localization and aggregation patterns of TDP43 were analyzed via fluorescence microscopy, and biochemical characterization of aggregates was performed using SDS‐PAGE, Western blotting, and immunocytochemistry.

**Result:**

Cellular stress activators induced distinct nuclear and cytoplasmic TDP43 aggregation patterns, indicating the involvement of multiple stress‐dependent pathways in TDP43 pathology. Broad HDAC inhibition triggered a time‐dependent mislocalization of TDP43 from the nucleus to the cytoplasm, suggesting that HDAC‐regulated acetylation is crucial for nuclear TDP43 retention. Furthermore, HDAC inhibition led to increased cellular acetylation, which promoted the formation of stable, SDS‐resistant TDP43 oligomers via disulfide‐linked aggregation. While phosphorylation was detected within these aggregates, our findings suggest that disulfide bonding plays a primary role in driving aggregation, rather than phosphorylation.

**Conclusion:**

Our findings highlight the critical role of acetylation‐mediated disulfide‐linked aggregation in TDP43 pathology and suggest that HDAC inhibition contributes to TDP43 mislocalization and stable oligomer formation. Targeting this pathway may provide a novel therapeutic strategy for TDP43 proteinopathies, including ALS and FTLD.

